# Microencapsulation of Neuroblastoma Cells and Mesenchymal Stromal Cells in Collagen Microspheres: A 3D Model for Cancer Cell Niche Study

**DOI:** 10.1371/journal.pone.0144139

**Published:** 2015-12-14

**Authors:** Pan Yeung, Hoi Shun Sin, Shing Chan, Godfrey Chi Fung Chan, Barbara Pui Chan

**Affiliations:** 1 Tissue Engineering Laboratory, Department of Mechanical Engineering, The University of Hong Kong, Pokfulam Road, Hong Kong Special Administrative Region, China; 2 Department of Adolescence Medicine and Paediatrics, Li Ka Shing Faculty of Medicine, The University of Hong Kong, Hong Kong Special Administrative Region, China; The University of Adelaide, AUSTRALIA

## Abstract

There is a growing trend for researchers to use *in vitro* 3D models in cancer studies, as they can better recapitulate the complex *in vivo* situation. And the fact that the progression and development of tumor are closely associated to its stromal microenvironment has been increasingly recognized. The establishment of such tumor supportive niche is vital in understanding tumor progress and metastasis. The mesenchymal origin of many cells residing in the cancer niche provides the rationale to include MSCs in mimicking the niche in neuroblastoma. Here we co-encapsulate and co-culture NBCs and MSCs in a 3D *in vitro* model and investigate the morphology, growth kinetics and matrix remodeling in the reconstituted stromal environment. Results showed that the incorporation of MSCs in the model lead to accelerated growth of cancer cells as well as recapitulation of at least partially the tumor microenvironment *in vivo*. The current study therefore demonstrates the feasibility for the collagen microsphere to act as a 3D *in vitro* cancer model for various topics in cancer studies.

## Introduction

Using 2D monolayer cultures of cancer cell lines as a simple model to study cancer research could be traced back to 1950s [1, 2]. However, similar to healthy tissues, tumor tissues are 3D entities with cells, extracellular matrix and other microenvironment. To date, it is generally agreed that the monolayer cell line culture poorly represents the *in vivo* phenomenon[3], where cell-cell and cell-matrix interactions exist, therefore limiting its ability to predict cancer cell response in reality [4].

In recent years, there is a growing trend for researchers to use *in vitro* 3D models in cancer studies [3, 5, 6] on topics such as tumor microenvironment [7], angiogenesis [8] and metastasis [9]. These models include spheroids [10] and microspheres [11, 12]. They support co-culture of multiple cell types, allows cell-cell and cell-matrix interactions, and thus better preserve the *in vivo* characteristics of tumor tissue. Some models are able to establish the structural diversity of tumor tissues with zones of proliferating, quiescent or necrotic cells [4]. The ability of these 3D models to include multiple niche factors enables partial recapitulation and close resemblance of the *in vivo* microenvironment of cancer cells [4, 13, 14], contributing to tumor disease modeling and personalized chemotherapy screening in the long run.

Tumors are not homogenous organs but very complex tissues involving various cell types including but not limited to cancer cells, cancer progenitor cells, endothelial cells, inflammatory cells and cancer-associated fibroblasts [3, 15–17]. Apart from the proliferating neoplastic parenchymal cells (cancer cells), the supportive stroma made up of cells of mesenchymal origin could account for half of the stromal mass [3]. The progression of cancer does not solely depend on cancer cells but also on the stromal cells residing in the tumor microenvironment [18, 19]. It has been shown that multipotent mesenchymal stem cells (MSC) reside in adult tissues [20, 21]. Even though whether these cells originate from bone marrow remains controversial but the close resemblance of MSC with pericytes along the blood vessels wall providing another appealing explanation [22, 23]. Growing evidences show that cancer associated stroma particularly fibroblastic cells accelerated tumor growth [3] and promoted a permissive microenvironment for cancer metastasis [24, 25]. Some findings indicate that mesenchymal stem cells (MSCs) would transit from bone marrow to tumor during tumor development [26–29]. Nevertheless, the role of MSC in tumorigenesis remains controversial [26, 30–33]. One well known notion is that, the heterotypic interaction between multiple cell types is necessary for accurate resemblance of *in vivo* responses. In order to achieve this goal, 3D models enabling interactions among multiple cell types are attractive in studying such complicated interactions.

We have previously established a collagen microencapsulation platform, which entraps living cells within a reconstituted nanofibrous collagen meshwork [34]. The collagen meshwork is biocompatible, providing a physiologically relevant microenvironment permissive to cell attachment, proliferation, migration and differentiation in a wide range of cells including MSCs [34–37], HEK293 cells [38], embryonic stem cells [39], chondrocytes [40], nucleus pulposus cells [41] and osteoblasts [42]. One major advantage of the collagen microencapsulation model is the fact that the template collagen meshwork supports matrix remodeling, which refers to simultaneous degradation and deposition of extracellular matrix, when culturing mature cells and differentiating stem cells in 3D. This strongly justifies its usefulness in acting as a model recapitulating the *in vivo* cellular microenvironment during structural and functional tissue formation. A second major advantage of the collagen microencapsulation is its controllable and miniaturized (hundreds of microns in diameter) size [34] that a micro-tissue consists of several hundred of cells enables the capability on economical, personalized and high throughput screening.

Neuroblastoma (NB) is a pediatric cancer accounting for 6% of all malignancies found in children [43]. NB microenvironment consists of extracellular matrix, stromal fibroblasts, vascular cells and immune cells [3]. Specifically, stromal fibroblasts have been shown to enhance tumor growth, angiogenesis and metastasis [44, 45]. Reports also show that co-culture of the neuroblastoma cells (NBCs) with other cell types would lead to significantly different behaviors. For example, non-contact co-culture of NBCs with hepatocytes lead to less apoptosis activity and higher VEGF expression [46, 47]. In another example, co-culture of NBCs with HUVEC reduced the detectability of the cancer cells to neutrophils [48]. Moreover, cross-talks between NBCs and Schwann cells have been shown to stimulate NB differentiation, reducing the aggressiveness of the tumor [49, 50] shedding lights on new therapeutic strategies. On the other hand, a few reports have shown that the presence of MSC would increase the invasiveness of neuroblastoma [27, 51, 52]. Though the role of MSC on neuroblastoma is not completely known, the presence of MSC does lead to behavioral change in NBCs. In the meantime, although all these studies have shown that NBCs actively interact with other cell types, these experiments are all conducted in 2D models. A 3D model permissive to NBCs growth and proliferation, as well as interactions with stromal cells has not yet been reported. In this study, we hypothesize that the co-microencapsulation of NBCs with MSCs in collagen microspheres will recapitulate, at least partially, the tumor microenvironment *in vivo*. Specifically, we aim to investigate the morphology, growth kinetics and matrix remodeling in the co-microencapsulation environment.

Neuroblastoma (NB) is a pediatric cancer accounting for 6% of all malignancies found in children [43]. NB microenvironment consists of extracellular matrix, stromal fibroblasts, vascular cells and immune cells[3]. Specifically, stromal fibroblasts have been shown to enhance tumor growth, angiogenesis and metastasis [44, 45]. Reports also show that co-culture of the neuroblastoma cells (NBCs) with other cell types would lead to significantly different behaviors. For example, non-contact co-culture of NBCs with hepatocytes lead to less apoptosis activity and higher VEGF expression [46, 47]. In another example, co-culture of NBCs with HUVEC reduced the detectability of the cancer cells to neutrophils [48]. Moreover, cross-talks between NBCs and Schwann cells have been shown to stimulate NB differentiation, reducing the aggressiveness of the tumor [49, 50] shedding lights on new therapeutic strategies. On the other hand, a few reports have shown that the presence of MSC would increase the invasiveness of neuroblastoma [27, 51, 52]. Though the role of MSC on neuroblastoma is not completely known, the presence of MSC does lead to behavioral change in NBCs. In the meantime, although all these studies have shown that NBCs actively interact with other cell types, these experiments are all conducted in 2D models. A 3D model permissive to NBCs growth and proliferation, as well as interactions with stromal cells has not yet been reported. In this study, we hypothesize that the co-microencapsulation of NBCs with MSCs in collagen microspheres will recapitulate, at least partially, the tumor microenvironment in vivo. Specifically, we aim to investigate the morphology, growth kinetics and matrix remodeling in the co-microencapsulation environment.

## Methods

### Neuroblastoma cell culture

Neuroblastoma cell line was a kind gift from Dr. NKV Cheung from Memorial Sloan Kettering Cancer Center, USA. Cells were cultured at 37°C in a 5% CO2 incubator in Dulbecco’s modified Eagle medium (DMEM) with high glucose (Gibco), with supplements of 10% Fetal Bovine Serum (Gibco), 1% penicillin streptomycin (Gibco), 1% glutar-max (Gibco).

### Mesenchymal stem / stromal cell (MSCs) culture

Human MSCs from bone marrow [53] were kindly provided by Prof. GCF Chan, Department of Paediatrics and Adolescent Medicine, The University of Hong Kong and cultured as monolayers as previously described [53]. The current protocol has been approved by the Combined Clinical Ethics Committee of the University of Hong Kong and Hong Kong West Cluster Hospitals of Hospital Authority. In brief, MSCs were cultured at 37°C in a 5% CO^2^ incubator in DMEM with low glucose (Gibco), with supplement of 10% Fetal Bovine Serum (Gibco), 1% penicillin streptomycin (Gibco) and 1% glutar-max (Gibco). The growth medium was replaced every 3–4 days. At around 80% confluence, hMSCs were isolated by trypsinization with 0.05% trypsin-EDTA (1X) (Gibco) briefly before re-suspending in full medium for subsequent experiments. Cells at P6 were used for subsequent experiments.

### Collagen microencapsulation

Cells were microencapsulated as previously reported [34]. In brief, hMSC and NBCs were cultured to sub-confluence and were then detached by treating NBCs and MSCs with 0.25% and 0.05% trypsin- EDTA(1X) (Gibco) for 5 minutes. NBCs and MSCs were mixed at different predetermined ratios (NBCs: 100, 80, 50, 20 and 0%) before microencapsulation. Rat tail type I collagen (Becton Dickenson Biosciences, Bedford, MA) was neutralized by 0.1N NaOH and diluted into different final concentrations (0.5, 1 and 2mg/ml). Cell mixtures were suspended in neutralized collagen solution to make up cell–matrix mixtures with different final cell densities (2.5x10e5, 5x10e5 cells/ml and 1x10e6 cells/ml, equivalent to 1250, 2500 and 5000 cells/5μl droplet, respectively). Liquid droplets of cell-matrix mixtures were dispensed onto a non-adhesive surface, which is UV-irradiated parafilm in a 90-mm diameter Petri dish (Sterilin, London, United Kingdom), and then incubated at 37°C with 5% CO^2^ for 45 minutes to induce gelation. Gelated collagen microspheres containing both NBCs and MSCs at predetermined ratios were gently flushed with a co-culture medium into a Petri dish for free-floating suspension cultures for different duration (7, 14 and 21 days). A total of 100 microspheres were cultured in each petri dish. The co-culture medium was mixed by NBC and MSC culture medium according to cell encapsulation ratio, replenished every 2–3 days.

### Measurement of the dimension of the cell-matrix microspheres

The temporal morphological change of the NBC-MSC-collagen microspheres was recorded under a phase contrast microscope up to 21 days. The diameters of approximately 10% of the microsphere populations were randomly selected and measured using an eye-piece micrometer.

### Growth kinetics of cells

Every 200 microspheres were encapsulated with 2.5e5 cells with different NBC: MSC ratios at day 0, and they were cultured for 7, 14, and 21 days. At each time point, 200 microspheres from each group were digested enzymatically by collagenase from Clostridium histolyticum (Sigma) at 200 units/ml for 45–60 min. Single cells suspensions were obtained by treating the digested aggregates with 0.25% trypsin-EDTA(1X) (Gibco) before numeration by trypan blue assay. The growth of cells in microspheres could then be calculated.

### Flow cytometry analysis on the proportion of NBCs

Single cell suspensions (1x10e6 cells) obtained from collagenase-trypsin digestion of the NBC-MSC-collagen microspheres were re-suspended in 500 μl of co-culture medium, incubated at room temperature for an hour to allow the recovery of cell surface protein expression, and were then fixed by 0.01% PFA for 15 minutes. Cells were then blocked by 2% Goat serum (Vector Laboratories) in PBS for 30 minutes before indirect staining of antibodies. To each sample, 1μl of mouse monoclonal antibody against Neuroblastoma (NB84a, abcam) in 2% Goat serum (dilution 1:100) was added. Isotype controls (normal mouse IgG antibody, Millipore) were performed at each time point. After staining at room temperature for 30 minutes, 1ml PBS was added to each tube to wash off the excess antibodies. After centrifugation at 2000 rpm for 5 min, the supernatant was removed and 0.5μl of Alexa Fluor 647 goat Anti-mouse secondary antibody (Invitrogen) in 2% goat serum (dilution 1:200) was added to each sample. After staining in dark at room temperature for 30 minutes, 1ml PBS was added to each tube to wash off the excess antibodies. After centrifugation at 2000 rpm for 5 min, the supernatant was removed and Cell pellets were resuspended and preserved in 500μl 1% PFA at a cell density not less than 4x10e5 cells/ml for flow cytometry analysis in FACSCanto II Flow Cytometer (BD Biosciences, Bedford, MA). 10,000 events of each sample were analyzed. Results were analyzed with Flowing Software 2.5.1.

### Histology and immunohistochemistry of NBC-MSC-collagen microspheres

NBC-MSC-collagen microspheres were fixed in 4% PFA at room temperature in dark for 30 minutes and were dehydrated using a serial gradient ethanol treatment before processing for paraffin sections of 5μm thickness. Routine hematoxylin and eosin (Sigma) staining was conducted to reveal the cell morphology in the microspheres. To evaluate the presence of NBCs, a primary antibody (ab49501, abcam) was used. Anti-mouse secondary antibody (BA-1000, Vector laboratories) was used in immunohistochemistry, followed by ABC staining, diaminobenzidine labelling, and counterstaining using hematoxylin. To evaluate the presence of type I collagen, a primary antibody (C2456, Sigma), was used. Anti-mouse secondary antibody (BA-1000, Vector laboratories) was used in immunohistochemistry, followed by ABC staining, diaminobenzidine labelling, and counterstaining using hematoxylin. To evaluate the presence of Matrix-metalloproteinase 9, a primary antibody (ab38898, abcam) was used. Anti-rabbit secondary antibody (BA-2000, Vector laboratories) was used in immunohistochemistry, followed by ABC staining, diaminobenzidine labelling, and counterstaining using hematoxylin.

### Data analysis and statistics

Quantitative results including microsphere dimension, cell numbers and NBC proportions were presented as mean ± standard deviations if not otherwise stated. Two-way ANOVA with appropriate post hoc tests were used to reveal statistically significant differences among different groups. The significance level was set at 0.05 and SPSS 19.0 (IBM, NY, USA) was used to execute to statistical analysis.

## Results

### Morphological characterization of NBC-MSC-collagen microspheres

**Fig 1:**
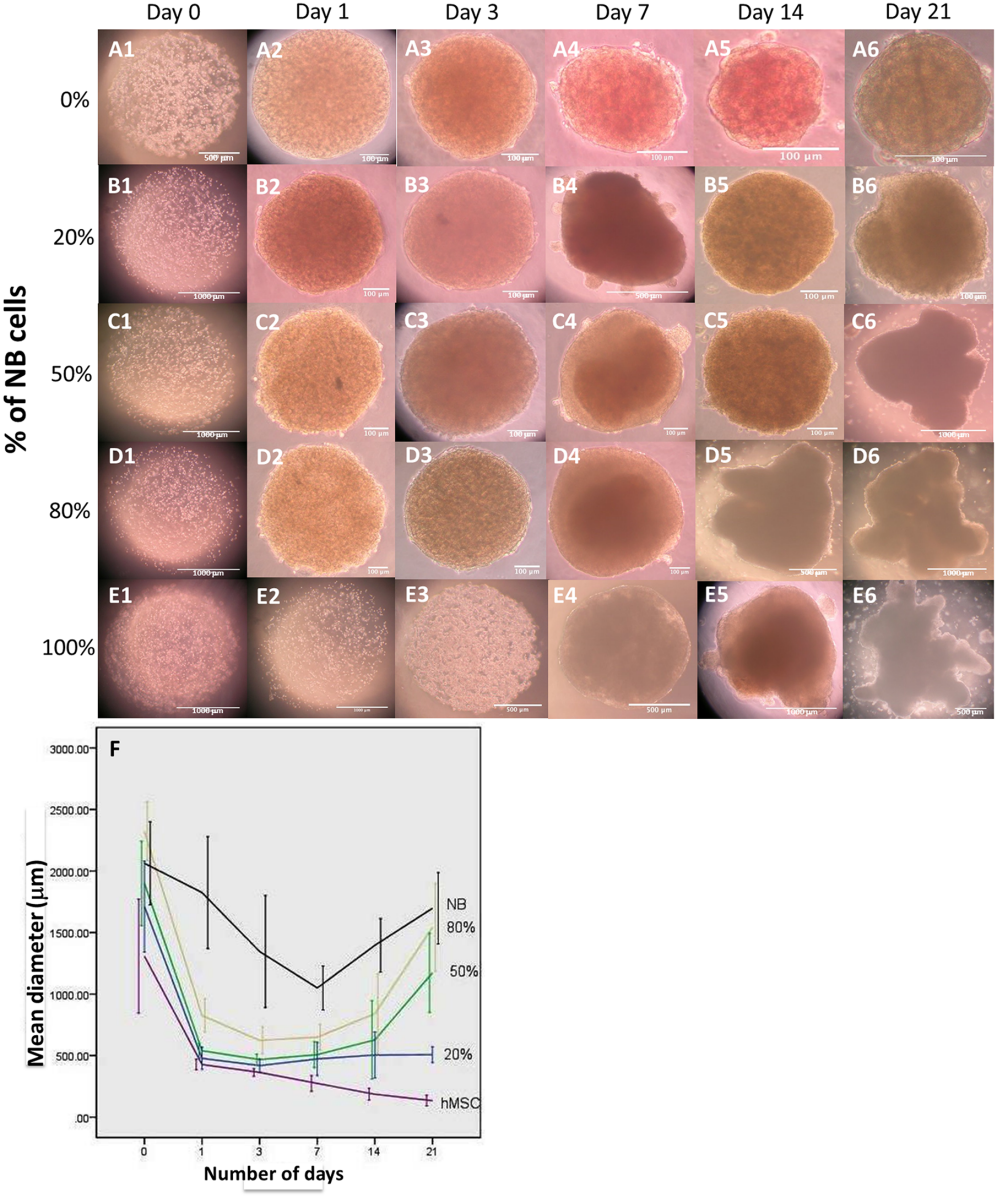
Gross appearance and dimension of neuroblastoma cell-mesenchymal stromal cell (NBC-MSC) co-encapsulated collagen microspheres. Microspheres with different percentage of NBCs and hence NBC:MSC ratios: (A): 0% NBC (100% MSC); (B): 20% NBC (80%MSC); (C): 50% NBC (50% MSC); (D): 80% NBC (20% MSC) and (E): 100% NBC (0% MSC) were cultured for different period of time: 0 (A1,B1,C1,D1,E1); 1 (A2,B2,C2,D2,E2); 3 (A3,B3,C3,D3,E3), 7 (A4,B4,C4,D4,E4), 14 (A5,B5,C5,D5,E5) and 21 (A6,B6,C6,D6,E6) days (scale bars: 100 μm: A2,A3,A4,A5,A6,B2,B3,B4,B5,B6,C2,C3,C4,C5,D2,D3,D4; 500 μm: A1,D5,E3,E4,E6; 1000 μm: B1,C1,C6,D1,D6,E1,E2,E5); (F): Line chart showing the temporal change in the dimension (mean±1SD) of the NBC-MSC-collagen microsphere populations during cultures.

Fig 1A1–1E6 shows the gross appearance of NBC-MSC-collagen microspheres in different groups. The spherical appearances were similar in the beginning of the culture but became diverse at later stage, as microspheres in groups with higher initial NBC proportion gradually lost their spherical shape and became irregular in conformation, suggesting overgrowth. During culture, tiny micro-masses in suspension were observed in NBC 100%, 80% and 50% groups after the first week. While in the NBC 20% group, observable masses appeared after the second week. The NBC 80% group had relatively larger amount of micro-masses than in other groups. There was no overgrowth micro-masses in the NBC 0% group (100% MSC group). Fig 1F shows the change in the dimension of the microspheres during culture. There was significant contraction of the microspheres in the first week for all groups, followed by rises in diameter in all cancer cell containing groups, suggesting the tumorigenic growth. In the meantime, fusion and aggregation of microspheres are observed. The graph also shows that the extent of contraction and enlargement is dependent on the NBCs content. All groups except the NBC 100% one dramatically decreased in size in the first day after encapsulation. Microspheres containing healthy MSCs only (NBC 0%) continuously drop in size over time. The NBC 20% group showed a constant dimension after the initial drop in size while groups with 50% or more NBCs started to increase in size after 7 days, suggesting rapid growth of the tumor cells. Two-way ANOVA showed that both the time factor (p<0.001) and the NBC: MSC ratio (p<0.001) significantly affected the dimension of the microspheres. Bonferroni post hoc tests showed that apart from the day1-day14 (p = 0.518) and the day3-day7 (p = 1.000) pairs, all other comparisons were statistically significantly different from others (p<0.001) while all NBC: MSC ratio groups were statistically significantly different from others (p<0.001).

### Morphological changes of NBC-MSC-collagen microspheres at different NBC: MSC ratios, time points, initial cell density and collagen concentration

**Fig 2:**
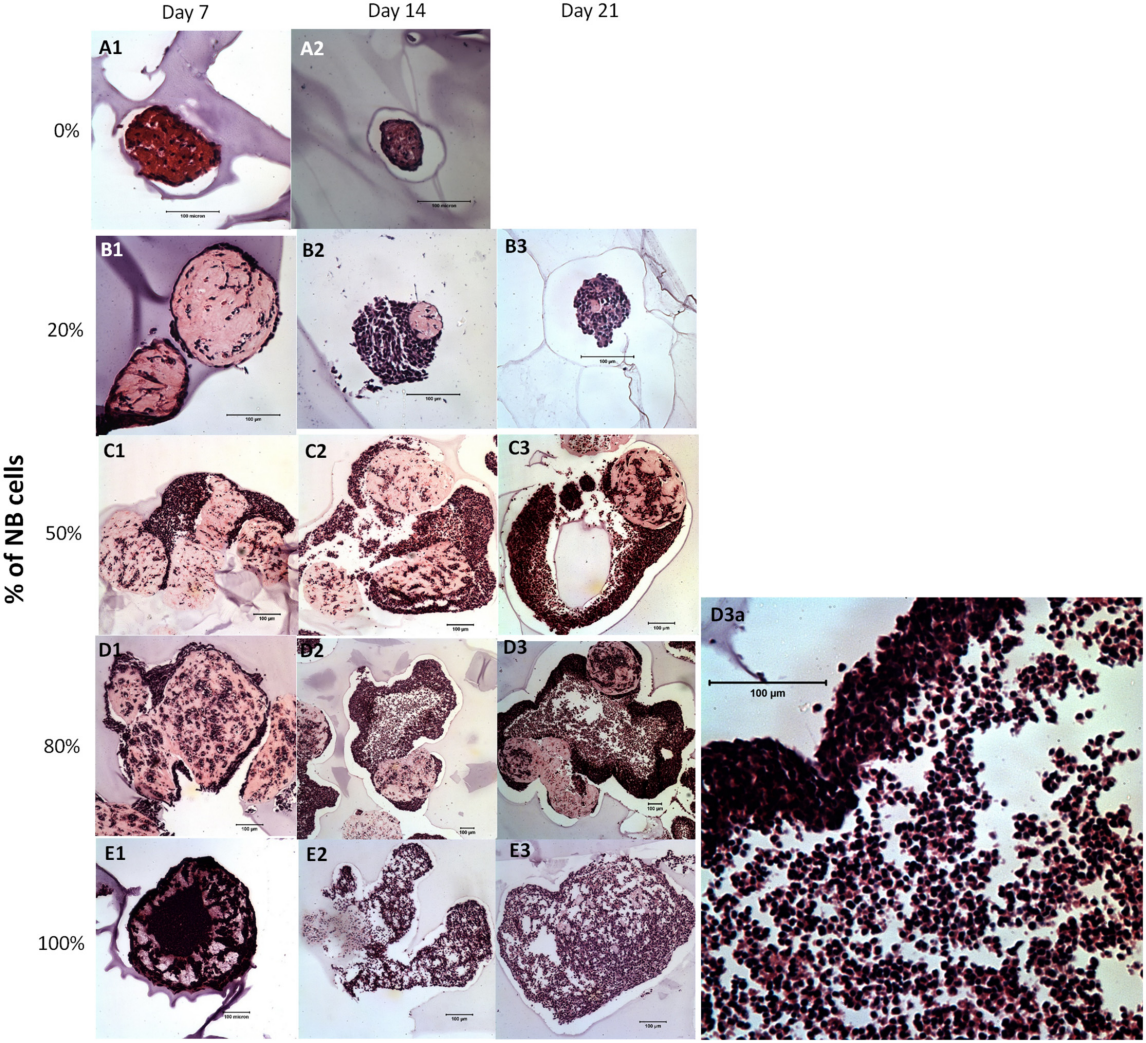
H&E staining showing the general morphology of NBC-MSC-collagen microspheres with different NBC proportions and at different time points. (A): 0% NBC (100% MSC); (B): 20% NBC (80% MSC); (C): 50% NBC (50% MSC); (D): 80% NBC (30% MSC); (E): 100% NBC (0% MSC). A1, B1, C1, D1, E1: day 7; A2, B2, C3, D2, E2: day 14; B3, C3, D3, D3a, E3: day 21 after culture (Scale bars: 100 μm).

**Fig 3:**
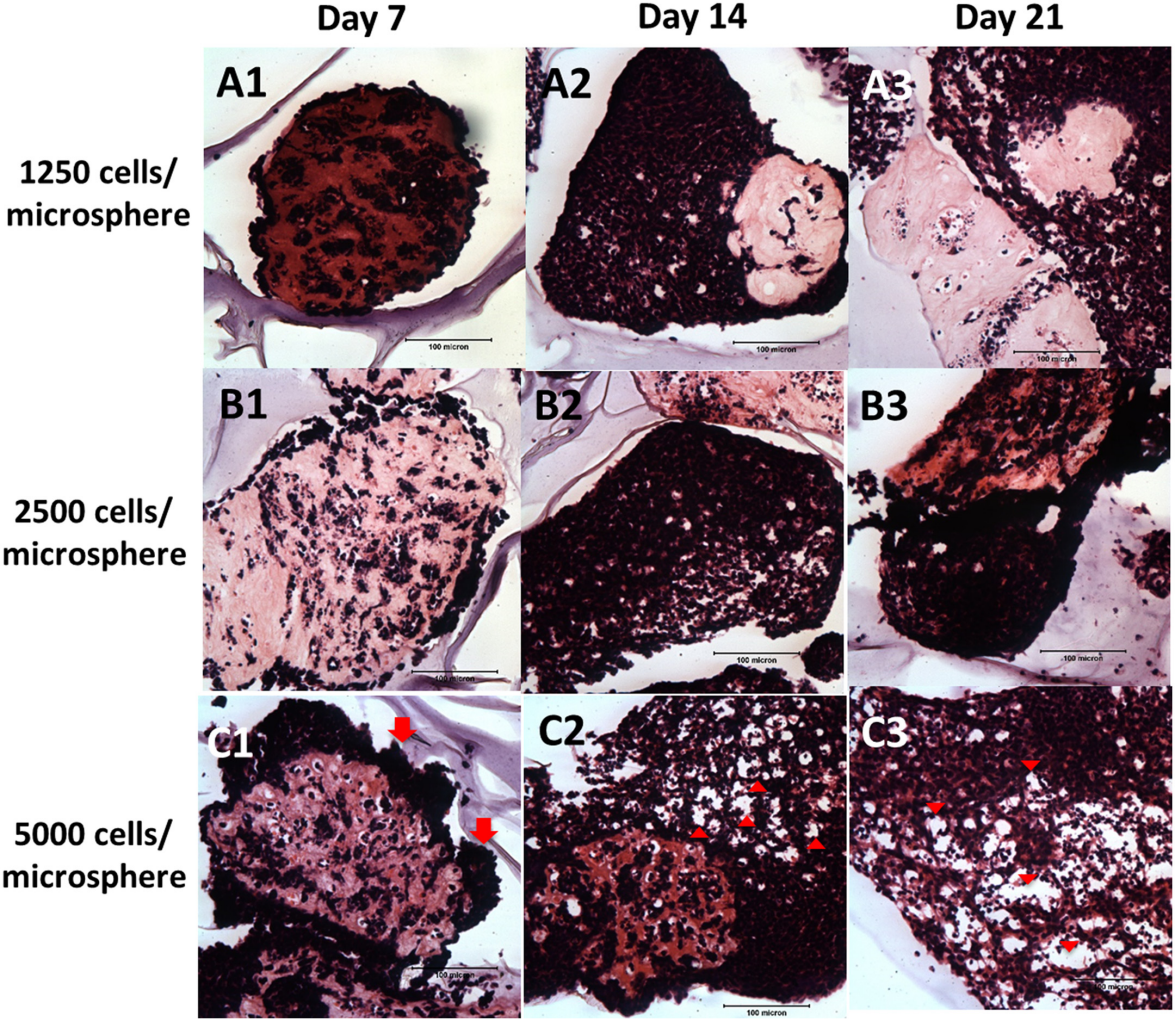
H&E staining showing the NBC-MSC-collagen microspheres at different encapsulation cell density. (A1-A3): 1250 cells/microsphere; (B1-B3): 2500 cells/microsphere; (C1-C3): 5000 cells/microsphere; 1: 7 days; 2: 14 days; 3: 21 days (scale bars: 100 μm).

Fig 2 shows the H&E staining of the NBC-MSC-collagen microspheres with different encapsulation ratios and at different time points during culture. The 0% NBC (pure MSC) group showed increasingly compact structures with randomly distributed MSCs for up to 2 weeks (Fig 2A1 and 2A2) while most of the microspheres showed complete attrition at 21 days of culture. Groups with increasing NBC: MSC ratios (20, 50 and 80%) (Fig 2B1–2D3) showed a similar trend that they segregated into two different layers of tissues. In brief, at 7 days of culture, a layer of cells packed at the surroundings of collagen microspheres in which cells with lower density were present (Fig 2B1, 2C1 and 2D1). In the 20% NBC group, there seems to be a higher ratio of cells with elongated morphology at 7 days (Fig 3B1) while relatively rounded cells with high cell density and low matrix density were dominant at later time points (Fig 2B2 and 2B3). Moreover, the micro-tissue masses were still largely spherical in shape. In the 50% NBC group, the shapes of the micro-masses were irregular and high density cell populations seems to outgrow the collagen microsphere and more cells invaded into the collagen microspheres at increasing time (Fig 2C1–2C3). In the 80% NBC group, a thin layer of high density cells was encapsulating the microspheres, which contains many cell clusters and “voids” at 7 days of culture (Fig 2D1). At 14 and 21 days, the structures were highly irregular in shape and highly porous with cells packed at high density throughout the structures while the collagen microspheres seems to be disintegrated (Fig 2D2 and 2D3). A magnified view of Fig 2D3 showed clearly the high density cell layer and the less dense region (Fig 2F). In the 100% NBC group, the structure was still spherical but highly compacted at 7 days (Fig 2E1) while the collagen microspheres were completely torn apart in later time points with highly porous and irregular structures with low cell density (Fig 2E2 and 2E3).

Fig 3 shows the H&E staining of the NBC-MSC-collagen microspheres at different encapsulation cell densities when NBC was fixed at 80%. At 1250 cells per microsphere, cells seems to be encapsulated within the collagen microsphere while a thin layer of cells covered the periphery of the microsphere at early time point (7 days) (Fig 3A1) but at later time points, irregular cellular outgrowths were found leaving only very few cells within the microsphere (Fig 3A2 and 3A3). At higher cell densities (2500 and 5000 cells per microsphere), obvious voids were found in the collagen microspheres with densely packed cells in irregular shaped outgrowths surrounding the microsphere at 7 days (Fig 3B1 and 3C1). At later time points, the outgrowths became larger and more porous (Fig 3B2, 3B3, 3C2 and 3C3).

**Fig 4:**
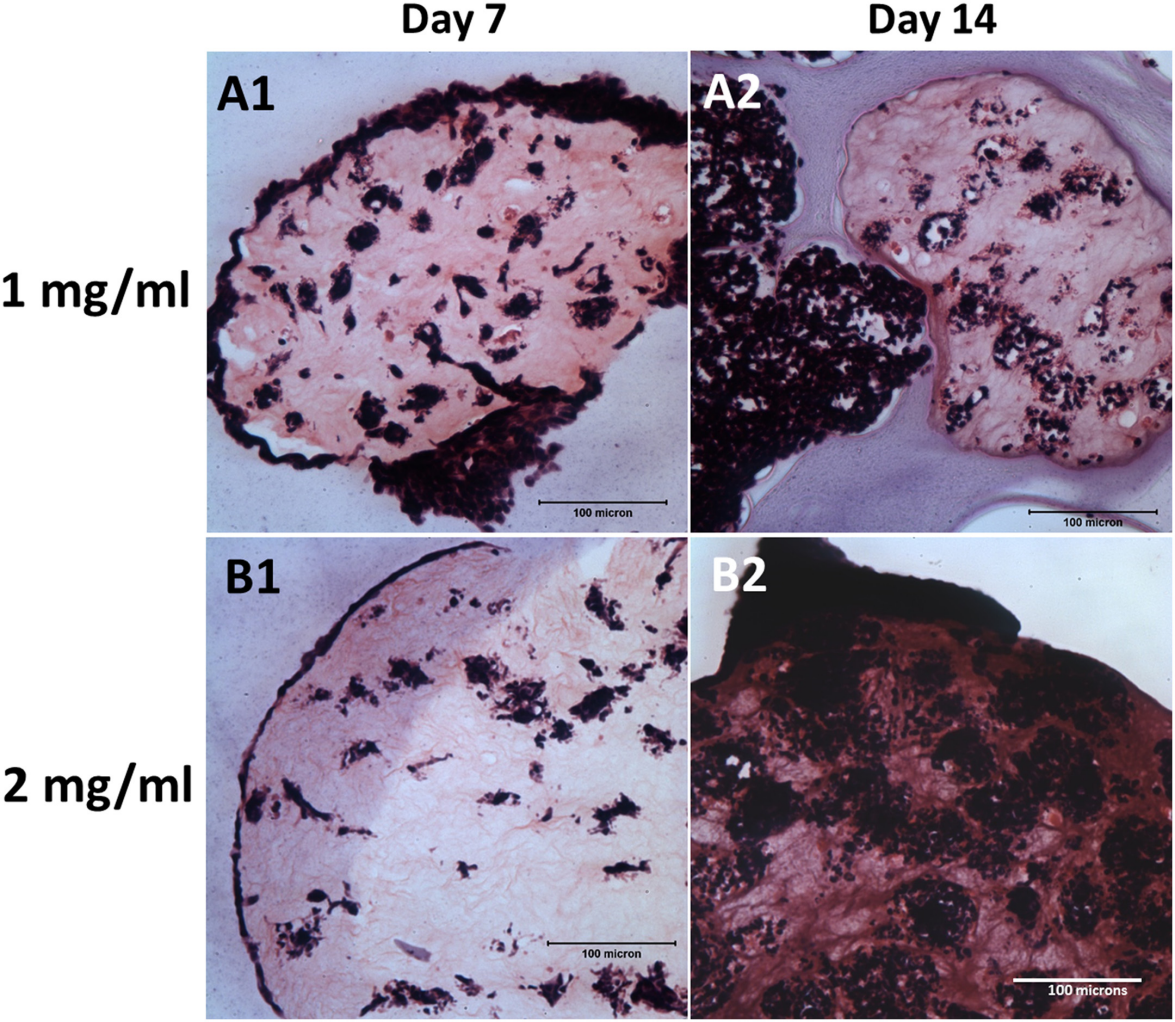
H&E staining of NBC-MSC-collagen microspheres at different collagen concentrations. (A): 1mg/ml; (B): 2mg/ml; 1: 7 days; 2: 14 days (scale bars: 100 μm).

Fig 4 shows the H&E staining of the NBC-MSC-collagen microspheres with different collagen concentrations. A higher collagen concentration seems to encapsulate the cells better within the microsphere while a thin layer of cells were growing at the periphery of the microsphere (Fig 4B1). At 14 days, irregular and massive outgrowth was found in the lower collagen concentration group (Fig 4A2) but dense colonies of cells and voids were found within the microspheres in the higher collagen concentration (Fig 4B2).

### Growth kinetics in NBC-MSC-collagen microspheres

**Fig 5:**
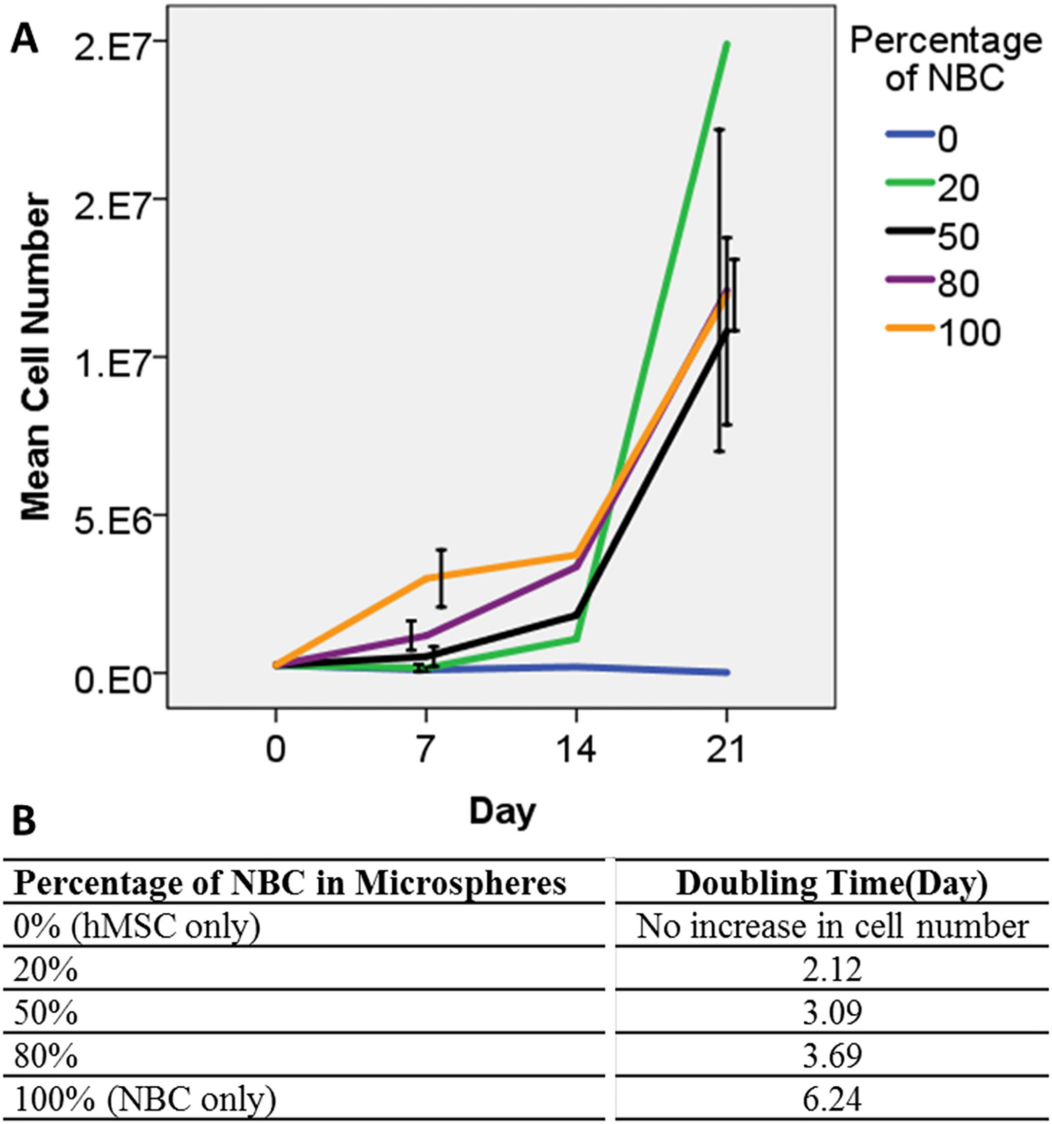
Growth kinetics of cells in co-cultured NBC-MSC-microspheres with different proportions of NBCs. At day 7, day 14 and day 21, 200 microspheres were digested to count the cell number. (A): Growth kinetic curves showing the changes in the number of cells over time at different encapsulating ratios (mean±1SD); (B): Population doubling time for different groups.

Fig 5A shows the line graph on the cell number in NBC-MSC-collagen microspheres with different NBC: MSC ratios at different time points during co-culture. Microspheres with 0% NBC, i.e. 100% MSCs showed a continuous drop in the cell number. On the other hand, all groups containing cancer cells showed a positive growth over time. The 20% group showed gradual increase in the first 2 weeks and all in a sudden the number dramatically increased exponentially on day 21. The 100% NBC group (0% MSC) showed significant increase in early cultures on day 7 than other groups but that did not provide this group any further growth advantage in later days. Similar trends were found in the 50% and 80% NBC: MSC groups. Two-way ANOVA showed that both the time factor (p<0.001) and the NBC: MSC ratio factor (p<0.001) significantly affected cell number. Bonferroni post hoc tests showed that 0% NBC (100% MSC) significantly different from all other groups (p< = 0.003). The 20% NBC group showed significant differences of 0% (p = 0.003), 80% (p = 0.033) and 100% (p = 0.008), respectively. The 50% NBC group, and the 80% and then 100% were not significantly different among themselves (p> = 0.342). The 100% NBC only showed significant difference from the 0% and 20% NBC: MSC ratios (p< = 0.008). Interestingly, starting with the same cell number, the 20% NBC group showed a much lower (~2 days) doubling time, which measures the time taken for the cell population to double itself, as compared with higher NBC:MSC ratio (Fig 5B). The 0% NBC (100%MSC) group showed no growth while the 100% NBC (0%) showed much lower doubling time (>6 days).

### Temporal change in the percentage of NBC in the microspheres

**Fig 6:**
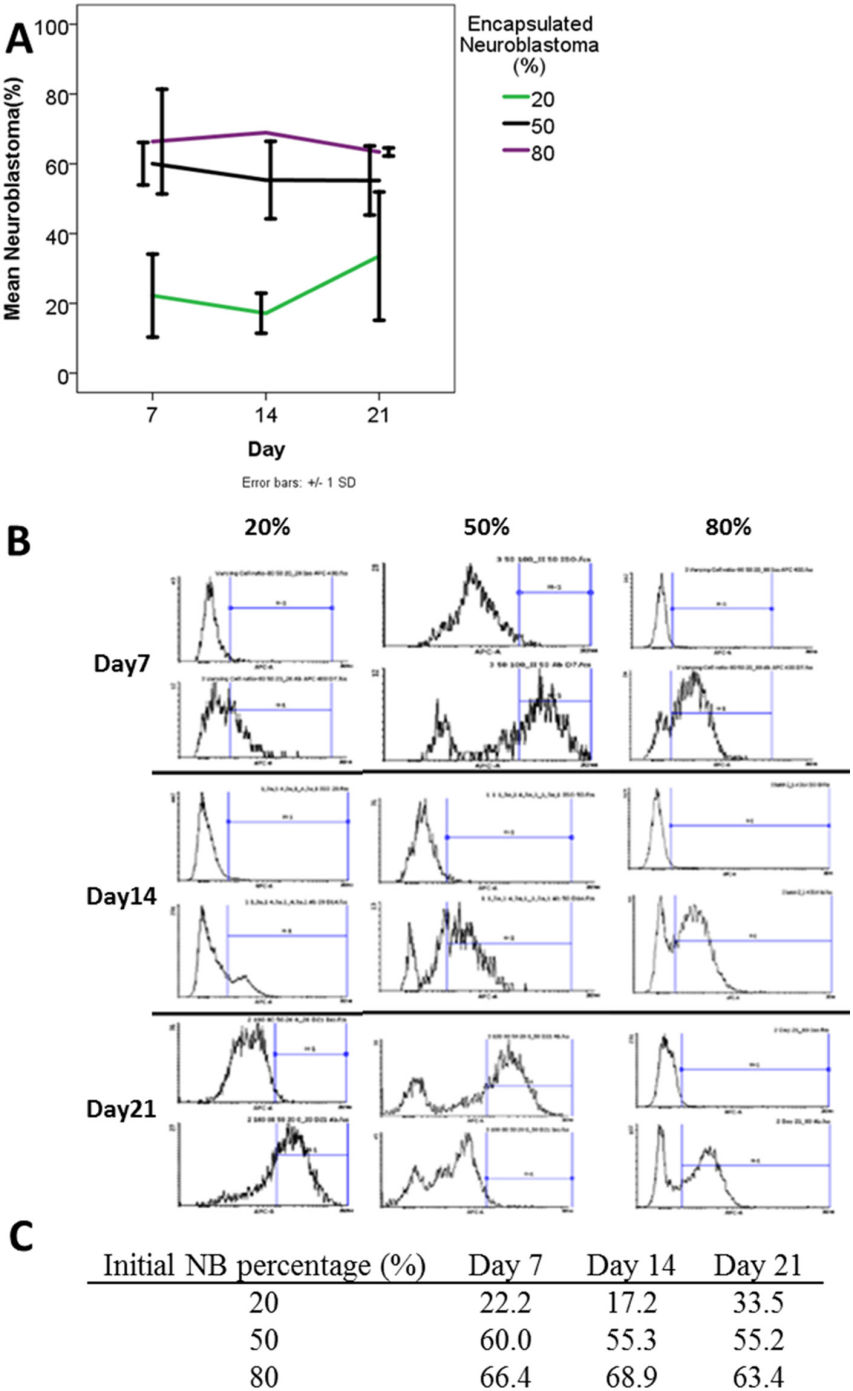
Percentage of NBCs in the NBC-MSC-collagen microspheres with different initial NBC: MSC ratios at different time points. (A): Error bar charts showing the percentage of NBCs determined via flow cytometry (mean±1SD); (B): Table showing the mean percentage of NBCs in different groups and at different time points; (C): Representative histogram showing cells stained with isotype control antibody and cells were stained with NB84a antibody in different groups at different time points.

Fig 6A shows the percentages of NBCs among different groups over time. In the 20% NBC group, the percentage of NBCs were maintained at around 20% at day 7 and 14 but dramatically increased to 33% at day 21 (Fig 6A). On the other hand, 50% and 80% NBC groups showed similar level of NBCs over time. Fig 6C shows the representative histogram of the flow cytometry-base enumeration of NBCs in different groups. Fig 6B shows the percentage of NBCs calculated from data obtained by flow cytometry. Two-Way ANOVA showed that NBC: MSC ratio factor (p<0.001) significantly affected the percentage of NBC overtime, but not the time factor (p = 0.85). Bonferroni post hoc tests showed that the 20% NBC group significantly different from 50% (p<0.001) and 80% (p<0.001) respectively. The 50% NBC group and the 80% were not significantly different among themselves (p = 0.366).

### Immunohistochemistry of Type I collagen

**Fig 7:**
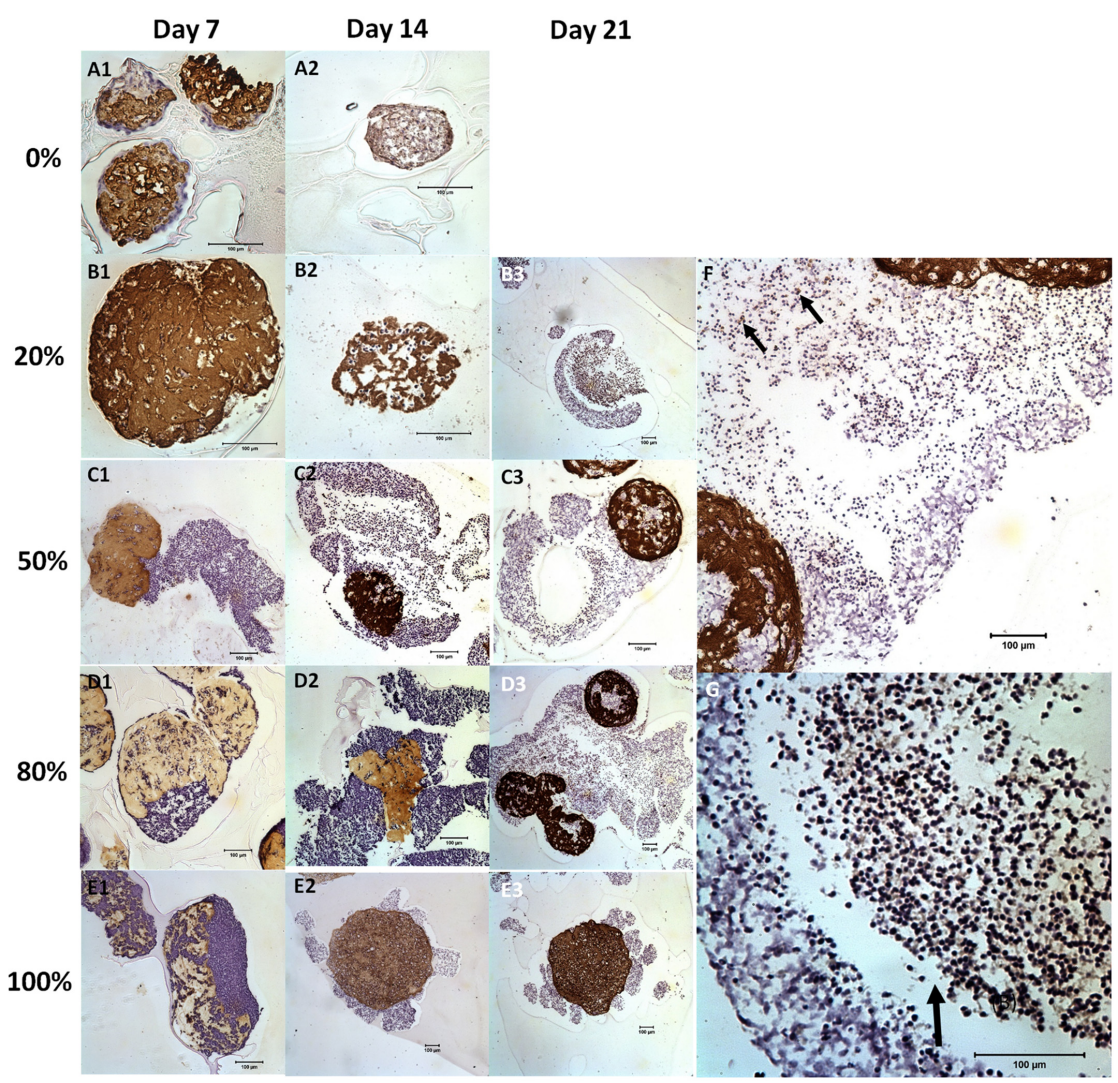
Immunohistochemistry of type I collagen of NBC-MSC-collagen microspheres with different NBC proportions and at different time points. (A): 0% NBC (100% MSC); (B): 20% NBC (80% MSC); (C): 50% NBC (50% MSC); (D): 80% NBC (30% MSC); (E): 100% NBC (0% MSC). A1, B1, C1, D1, E1: day 7; A2, B2, C3, D2, E2: day 14; B3, C3, D3, D3a, E3: day 21 after culture (Scale bars: 100 μm).

Fig 7 shows the immunohistochemical analysis of collagen type I in the NBC-MSC-collagen microspheres. In 0% NBC (100% MSC) group, the type I collagen positive microsphere remained spherical although it becomes more porous at 14 days (Fig 7A and 7B). In the 20% NBC groups, the microsphere was intact on day 7 but started to be decomposed on day 14 and was almost completely decomposed on day 21 (Fig 7B2 and 7B3). For the 50% NBC group, the microspheres were still intact at day 7 (Fig 7C1) but more porous at later time points (Fig 7C2 and 7C3). This trend was similar in the 80% NBC group (Fig 5B). Type I collagen expressing cells, which are likely to be MSCs, were mostly confined within the microspheres although some were identified occasionally in the outgrowth outside the microspheres (Fig 7F and 7G).

### Immunohistochemistry of MMP9

**Fig 8:**
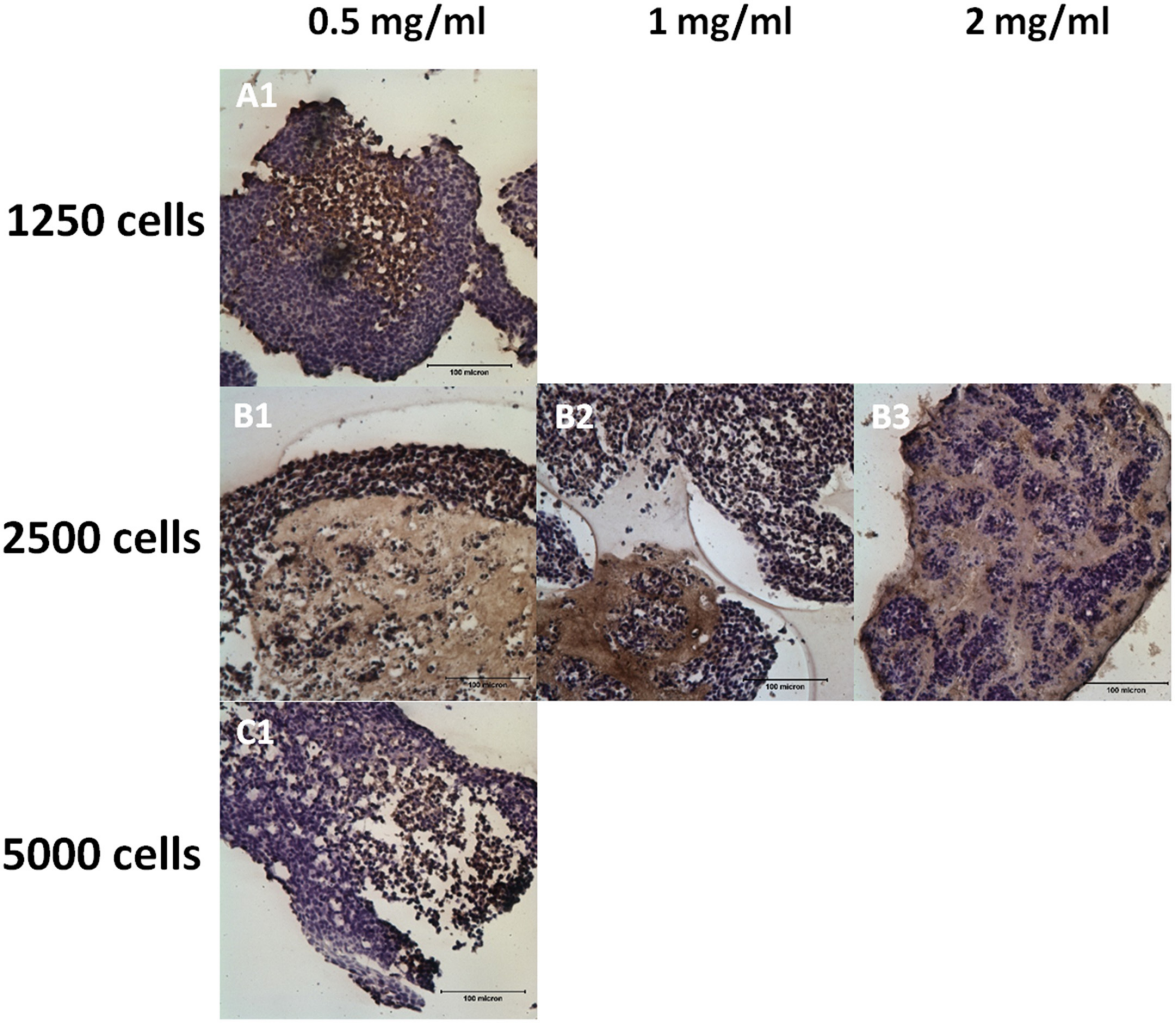
Immunohistochemistry of MMP9. (A): 5000 cells/ml; (B): 2500 cells/ml; (C): 1250 cells/ml; 1: 0.5 mg/ml; 2: 1 mg/ml; 3: 2mg/ml (scale bars: 100 μm).

Fig 8 shows the immunohistochemical staining of MMP9 of the NBC-MSC-collagen microspheres with fixed 80% NBCs and with different cell densities and collagen concentrations. The collagen regions showed positive staining while MMP9-expressing cells were found both in the out-growth and the peripheral layers of cell masses (Fig 8A1, 8B1, 8B2 and 8C1).

## Discussion

### In vitro cancer models

In order to accelerate preclinical drug screening and to achieve personalized medicine in the future, the development of disease or patient-specific in vitro model are of great importance[54]. As discussed above, conventional 2D monolayer models are being criticized for their non-physiological culture environment and they are not able to recapitulate the in vivo condition, therefore producing unreliable data. With the advance in field of tissue engineering, the use of an in vitro three dimensional model in mimicking tumors is becoming more popular. [55–57]. An important issue in fabricating an in vitro model would be the choice of biomaterials, which could be of natural origin or artificially synthesized. Numerous studies have shown promising results by using natural biomaterials such as collagen, fibrin, Matrigel[58] and hyaluronic acid. With their ready availability, ease of use and high bioactivity, they are popular and attractive choices for researchers. Collagen has an excellent biocompatibility and negligible immunogenicity. These properties enable scientists to use collagen as an appropriate material for in vitro cancer model. However, the ease of degradation, undefined matrix composition and weak mechanical properties are their problems. In addition, high batch-to-batch heterogeneity in composition makes comparison between different studies very difficult. On the other hand, synthetic materials such as PA, polyester, PEG possess tunable parameters and permit more precise control of material properties[59], leading to less complexity, high reproducibility and comparability between different studies. Yet some of them are toxic and they require more sophisticated processing steps before they can be used for modelling. PEG-based hydrogel, for instance, has non-fibrillar structure and requires either physical or chemical cross-linking processes [60–62]. These would affect the cellular behavior and the ease of use when compared to natural polymers like collagen. In fact, a universal biomaterial suitable for all disease models does not exist. Researcher should pick the one that fit for their particular interest of study.[56–58, 62] In recapitulating the in vivo tumorigenesis, a few features should be present in the model, including limitless proliferation, angiogenesis, invasion and metastasis.[57] Our study showed that collagen microspheres recapitulate several unique features of 3D tumor model such as irregular tumor outgrowth, epithelial-mesenchymal structures and tumor invasion, and vascular spaces. In the future, our group would further develop the collagen microsphere techniques in recapitulating other in vivo features, especially tumor angiogenesis which is critical in constructing a valid cancer models[55, 63, 64], by co-encapsulating endothelial cells.

### MSC provides a stromal niche supporting NBC growth

MSCs alone cultured in collagen microspheres maintain their viability without significant change in cell number. This reveals that MSC is a normal healthy adherence-dependent cell type that normally will not proliferate in 3D constrain. On the contrary, when NBCs were cultured in collagen microspheres, they rapidly proliferate and grow in an uncontrollable manner with irregular shaped outgrowth. This also reveals the tumorigenic nature of NBCs. When co-culturing these two cells types within the same microsphere, NBC growth is further facilitated by the additional reduction in the doubling time. Moreover, upon addition of 80% of MSC into the NBCs, the proportion of NBC increased from 20% to >35% in the microspheres. These strongly suggest that MSC provides a conductive niche facilitating NBC growth. MSCs have multiple differentiation potential that they can be stimulated to commit to multiple lineages when triggered by different signals or placed in different niches [65]. MSCs exhibit fibroblastic phenotype and may therefore provide a cancer-associated fibroblastic niche as reported [66, 67]. Cancer associated fibroblasts may secrete growth or signaling factors such as Wnt, BMP and Ephrin to affect cancer growth [68]. Similarly, MSCs are known to secrete a lot of soluble signals such as growth factors and chemokines [52, 69–71] that may also contribute to the supportive stromal niche for cancers with enhanced growth and metastasis. Fibroblasts and MSC may deposit and maintain their ECM such as type I collagen [72] and secrete growth factors including VEGF and TGF-β [73] to support the tumor growth. Another interesting observation of the current study is that the total number of cells in the microspheres initially containing 80% NBCs increased for more than 50 fold but the final NBC proportion was only maintained at around 60%, suggesting that growth of non-cancerous cells was stimulated. These cells are likely to be niche cells including stromal fibroblasts and endothelial cells, etc. Nevertheless, further studies are warranted.

### The co-encapsulation microsphere model mimics the in vivo characteristics of tumor tissues

The current 3D model mimics the *in vivo* characteristics of tumor tissues in manifold. First, Neuroblastoma represents a cancer of epithelial cells while MSCs in collagen provides a stroma-like environment. This mesenchymal-epithelial tissue structure was revealed by the spontaneously segregated dual-layer tissue structures. Moreover, is characterized by Homer Wright Pseudorosettes in histology [74]. In the current model, such “rosettes-like” morphology was observed occasionally (S1 Fig), suggesting that the current model mimics the *in vivo* tumor tissue well. Whether different fabrication parameters including the time points, ECM factors, cell factors and cell ratios can recapitulate different stages or subtypes of neuroblastoma deserves further investigation. Second, NBCs growth resulted in the formation of irregular tissue outgrowths, representing tumorigenicity and uncontrolled growth of cancer cells. Third, this model can be used to study EMT processes such as cancer metastasis, which may be due to the loss of epithelial adhesion molecules between cancer cells [75]. NBCs have been found to transmigrate or invade through the collagen microsphere or stroma-like tissues. This enables future studies on various cell types, soluble factors and matrix factors in affecting the EMT or the metastasis process, providing a model for basic cancer cell biology studies. For example, matrix metalloproteinase (MMPs) are important enzymes manipulated by cells to remodel the ECM and is also the important mediators of metastasis as cancer cells recruit these enzymes to make way for their invasion into matrix-rich mesenchymal cells [76]. MMP9 is reported to be closely associated with development of metastasis [77–79]. Our results showed that MMP9-expressing cells are usually found on the periphery of the microspheres or within the tissue micro-mass pinched off from the microspheres but not in the central region of the collagen microspheres. This might indicate that those actively migrating cells secrete MMP9 to facilitate their matrix digestion during invasion [80, 81]. Fourth, the collagen microsphere system may mimic the *in vivo* tumor tissue by providing a hypoxic environment. Our previous study showed the expression of HIF1α in ESC-derived chondrocytes cultured in the collagen microsphere platform [39] suggesting that cells at the center of the microsphere may sense hypoxic signal. Fifth, at 14 and 21 days and high cell densities, there were a lot of “voids” in the 3D tumor-like tissue or in another word, the tissue becomes very “porous”. This characteristic may be an analogous feature of the “leaky” structure of tumor [82], which refers to the formation of vascular spaces or lumens.

### Limitations of the current model

Tumor tissue has a unique feature in vascularization that the high metabolic demands of the cancer cells usually triggers vascular structure changes to achieve the purpose of enriching vascular nutrient supply. However, the current model does not include blood vessel cells such as endothelia cells and hence vasculature. Further studies including endothelial cells to form a multi-cellular tumor model warrants investigation. Preliminarily, we demonstrate the feasibility to use quantum dots of different fluorescence properties to label multiple types of cells including NBCs, MSCs and human umbilical cord derived endothelial cells (HUVEC) for a tri-culture model of the tumor niche (S2 Fig). Second, unlike NBC, MSC has no specific marker, therefore it is difficult to estimate its number in the population. As a result, we can only use flow cytometry to detect the number of NBC and calculate the number of non-NBC which may consists of MSCs, MSC differentiated cells like fibroblasts and endothelial cells, or even NBC differentiated cells. Third, our study demonstrates the capability to use collagen microencapsulation to reconstitute stromal microenvironment in 3D neuroblastoma model and investigates the tumor growth promoting effects of the stromal supportive niche. Nevertheless, answering particular cancer related biological questions and using it for drug screening have not been covered by the current report but would be the objectives of our future studies.

## Conclusion

Presence of MSCs in the 3D NBC model further promotes NBC growth with decreased doubling time and increased percentage of NBC at certain NBC: MSC ratio, suggesting that the presence of MSCs indeed serve as a supportive stromal niche. Moreover, the current model recapitulates several unique features of 3D tumor model such as irregular tumor outgrowth, epithelial-mesenchymal structures and tumor invasion, and vascular spaces, co-encapsulating NBCs and MSCs in 3D collagen microsphere therefore represent a potential 3D model for cancer niche studies.

## Supporting Information

S1 Fig Immunohistochemistry of NB84a as a marker for NBC in NBC-MSC-collagen microspheres. (scale bar: 100 μm).
(TIF)

S2 Fig Fluorescent images of NBC-MSC-HUVEC-collagen microspheres. NBC, MSC and HUVEC were labelled with qantum dots (Qdot 585, Qdot 655) and fluorescent dye (PKH67). NBC (Red: Qdot 585), MSC (Green: PKH67) and HUVEC (Blue: Qdot 655). (A): Day 7; (B): Day 14; (C): Day 21. (scale bars: 100 μm).
(TIF)
