# Supplementary figures and images for: Microencapsulation of Neuroblastoma Cells and Mesenchymal Stromal Cells in Collagen Microspheres: A 3D Model for Cancer Cell Niche Study
Source: PLoS One. 2015 Dec 14;10(12):e0144139. doi: 10.1371/journal.pone.0144139 (PMC4682120; doi:10.1371/journal.pone.0144139)

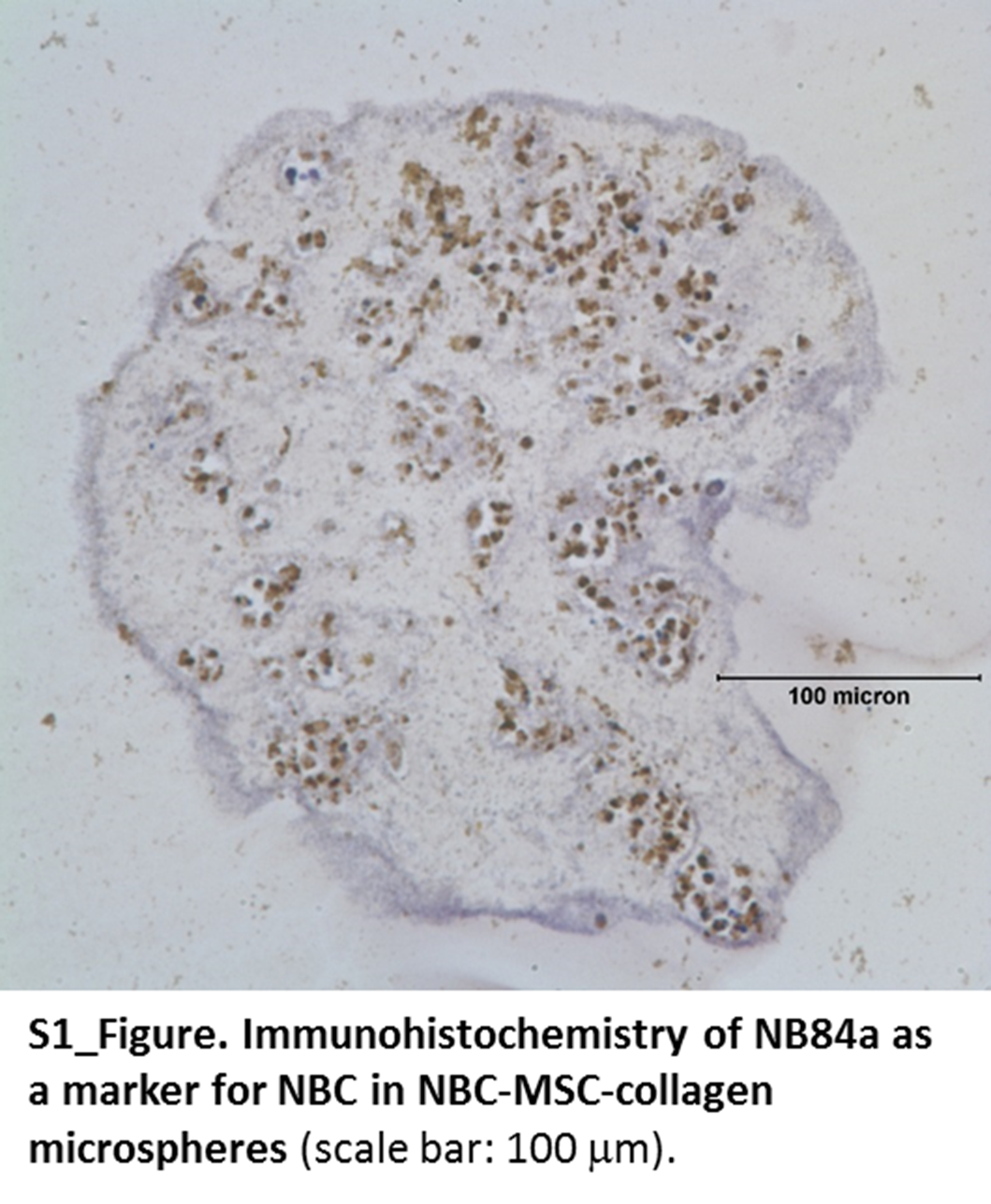

Supplement: S1 Fig — (scale bar: 100 μm). (TIF) [file pone.0144139.s001.tif]

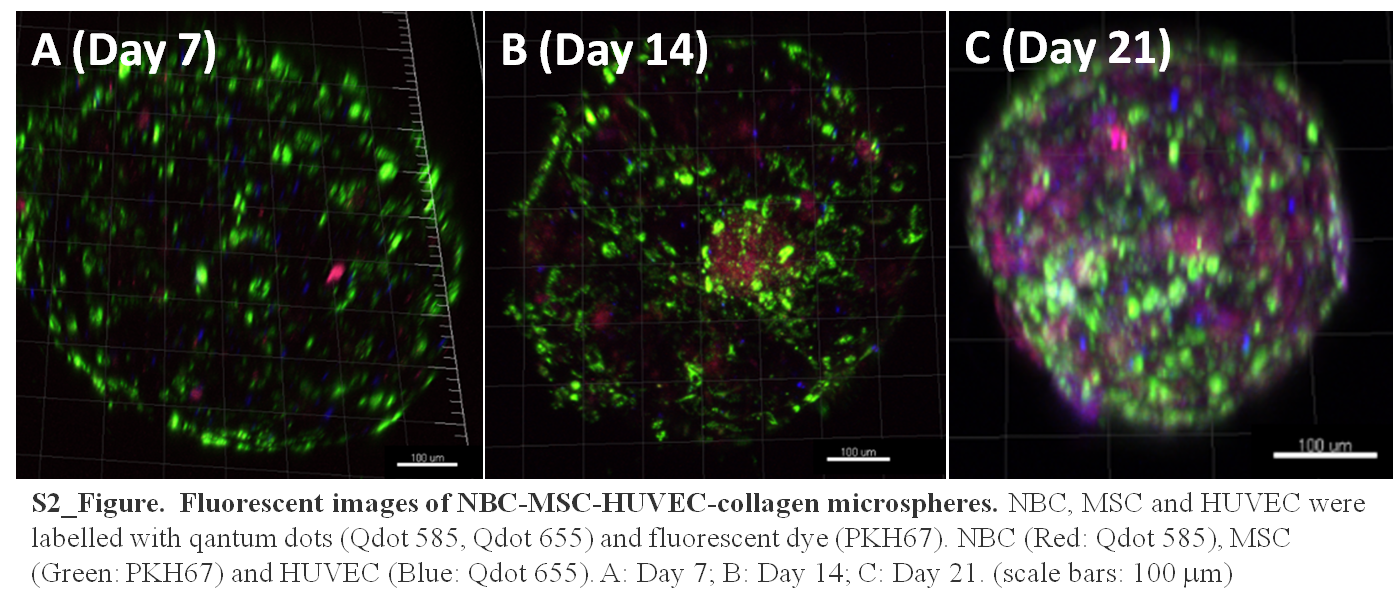

Supplement: S2 Fig — NBC, MSC and HUVEC were labelled with qantum dots (Qdot 585, Qdot 655) and fluorescent dye (PKH67). NBC (Red: Qdot 585), MSC (Green: PKH67) and HUVEC (Blue: Qdot 655). (A): Day 7; (B): Day 14; (C): Day 21. (scale bars: 100 μm). (TIF) [file pone.0144139.s002.tif]
